# Processed foods available in the Pacific Islands

**DOI:** 10.1186/1744-8603-9-53

**Published:** 2013-10-25

**Authors:** Wendy Snowdon, Astika Raj, Erica Reeve, Rachael LT Guerrero, Jioje Fesaitu, Katia Cateine, Charlene Guignet

**Affiliations:** 1 C-POND, Fiji National University and Deakin University, C/O College of Medicine, Nursing & Health Sciences, FNU (Tamavua Campus), Private Mail Bag, Suva, Fiji; 2 C-POND, College of Medicine, Nursing & Health Sciences, FNU (Tamavua campus), Private Mail Bag, Suva, Fiji; 3 Independent researcher, Apia, Samoa; 4 College of Natural and Applied Sciences, University of Guam, UOG Station, Mangilao 96923, Guam; 5 Nutrition and Dietetics Unit, Ministry of Health, , Republic of Nauru; 6 RESIR, Noumea, New Caledonia; 7 Independent consultant, Noumea, New Caledonia

**Keywords:** Pacific Islands, Processed foods, Nutrition

## Abstract

**Background:**

There is an increasing reliance on processed foods globally, yet food composition tables include minimal information on their nutrient content. The Pacific Islands share common trade links and are heavily reliant on imported foods. The objective was to develop a dataset for the Pacific Islands on nutrient composition of processed foods sold and their sources.

**Methods:**

Information on the food labels, including country of origin, nutrient content and promotional claims were recorded into a standardised dataset. Data were cleaned, converted to per 100 g data as needed and then checked for anomalies and recording errors.

Setting: Five representative countries were selected for data collection, based on their trading patterns: Fiji, Guam, Nauru, New Caledonia, and Samoa. Data were collected in the capitals, in larger stores which import their own foods. Subjects: Processed foods in stores.

**Results:**

The data from 6041 foods and drinks were recorded. Fifty four countries of origin were identified, with the main provider of food for each Pacific Island country being that with which it was most strongly linked politically. Nutrient data were not provided for 6% of the foods, imported from various countries. Inaccurate labels were found on 132 products. Over one-quarter of the foods included some nutrient or health-related claims.

**Conclusions:**

The globalisation of the food supply is having considerable impacts on diets in the Pacific Islands. While nutrient labels can be informative for consumers looking for healthier options, difficulties still exist with poor labelling and interpretation can be challenging.

## Background

The Pacific Island region is in the midst of a nutrition and epidemiological transition [1], experiencing under and over nutrition, infectious and non-communicable diseases. Extensive changes have been occurring in diets in the region, with an increasing reliance on imported foods and declining food self-sufficiency [2]. Imported and processed products such as rice, bread and noodles are increasingly replacing traditional staples; meat products are replacing fish; and sugary products are replacing traditional snacks such as fruits [1]. Available data suggests that food energy availability and fat/oil availability have increased considerably in recent decades [2] and the increasing imports in food parallels increasing energy density [3] and consumption.

There is considerable concern in the region regarding the effects of changing diets on health [4]. There is a need to better understand the supply of food in the region, sources, and nutrient content, to better guide interventions to improve diets. Existing Pacific Island Food Composition tables [5] are the only food composition tables in the region and include many of the foods and dishes unique to the region. However, they include only a limited amount of information on processed foods, and this is all composite data (average of the analysis of multiple samples), mostly sourced from other tables globally. Considerable differences have been highlighted elsewhere in the levels of sodium in processed foods [6], and the use of composite data can be misleading when identifying dietary problems. Data on processed foods would therefore be a useful complement to the existing tables. For the purposes of this study, processed foods were defined as pre-packaged food and beverages which had more than one ingredient. Given the differences in nutrients found between similar processed foods, data on their contents would also be of value in work with the food industry on reformulation.

As is seen elsewhere in the world, the globalisation of food supply has led to foods being shipped long distances and to significant changes in food availability within countries [7]. A study in 2008 [8] in the region identified that food products from 26 countries were available, with foods from Australia, New Zealand and China being most commonly found. Some of the same products were found in multiple countries in the region. Development of good quality food safety and labelling legislation still needs more work in the Pacific Island region [4], with many countries still not requiring nutrient information panels on food labels. Processed foods therefore only need to comply with the legislation in each country in regard to nutrition labelling. Those countries that have updated their food regulations more recently, still struggle with enforcement issues, which means that it is not uncommon to see unsuitably labelled products.

This paper reports on a study conducted in the region to collect nutritional information on processed foods and discusses the findings and implications for food supply, diets and health.

## Methods

The 21 Pacific Island countries share trading and shipping links, and in particular certain countries are 'hubs’ for trade. Providers of processed foods were primarily with countries within the region, or countries with which there were strong economic links such as France, Australia, New Zealand and the United States (US). On this basis, and due to resource limitations, five countries were identified which were considered by the authors to be hubs for trade or to have unique supply chains, thus representing the range of products available in all the Pacific Island countries. These were: Fiji, Samoa (independent state of, formerly known as Western Samoa), Guam, New Caledonia and Nauru.

To ensure uniformity of data collection across the sites, a standard protocol was developed based on the protocol of an existing global collaboration on nutrients in processed foods [9] – Food Monitoring Group. In each site (except Nauru), stores to be included in the data collection were identified in the capital city based on their size (at least 6 checkouts) or their number of outlets (three or more). In Nauru, the larger stores which imported their own goods were included. Permission was sought as needed from Ministries of Health and store-owners. Research assistants in each site collected data following a standard protocol from processed packaged foods (containing more than one ingredient) in the designated stores using forms provided. Data were then transferred to excel spreadsheets [9]. Standard food categories were used to classify food products into types to assist with later comparison [9]. Email and phone calls were used to clarify issues of design prior to commencing data collection. Data collection occurred in all sites between July and December 2011.

Label data on nutrient content, recommended serving size, health and other claims were recorded, along with brand name, manufacturer and country of manufacture. Nutrient data were required per 100 g, where information was provided per serving, this was recorded and values per 100 g calculated.

Data were checked for any anomalies or unexpected values by the principal author, and labels rechecked by repeat visits to the relevant stores for verification. Data of questionable accuracy was identified by the researchers, through comparison with similar products in the dataset and also by ensuring that added values of macronutrients did not exceed 100 g per 100 g. Data from all sites were then merged to produce a regionally-relevant dataset. Data collected in New Caledonia was primarily in French, while it was in English in the other sites. Analysis was undertaken of the dataset by country, and also at a regional level in regard to types of products, country of origin, extent of nutrition labelling and variety available. Additionally for selected popular products (product categories with highest range of products), fat and sugars levels were compared.

## Results

In total data from 6041 foods were collected. Table 1 shows the number of products included in the study from each site as well as information on labelling and country of production. Guam had the largest range of products followed by Fiji, while Nauru had the smallest range. One hundred and fifteen foods were found in more than one country.

**Table 1:** **Overview of the number of products by country** (**including labelling and product information**)

	**Fiji**	**Guam**	**Nauru**	**New Caledonia**	**Samoa**
Number of products recorded	1443	2105	234	1333	926
Number of products with no nutrient data	69	42	0	193	51
Predominant three countries of manufacture (and % of products from each)	Aus – 26% Fiji – 21% NZ – 13%	USA – 59% Philippines – 4.7% Japan – 3%	Aus- 56% Malaysia-7.7% Fiji- 7.2%	France – 51% New Caledonia -13 % Aus – 7.5%	NZ- 41% USA – 17% Aus – 9%
Number of products produced within country	309	6	0	178	31

**Figure 1:**
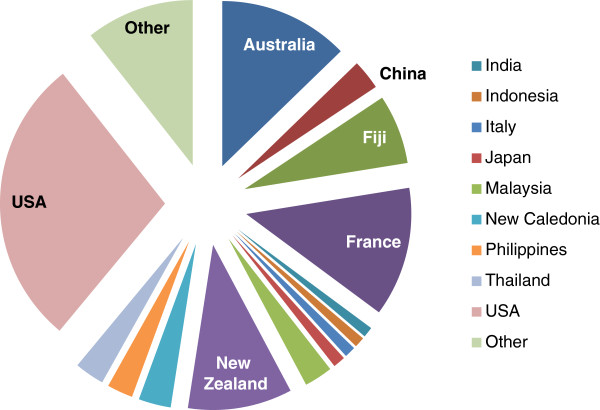
Countries of manufacture of processed foods sold in Pacific countries.

The country of origin of the food products mirrored the country with which they had the strongest economic and political ties, for example 59% of the products found in Guam were from the US, whereas for Nauru a similar percent of products were from Australia – Nauru uses the Australian dollar as currency, and Guam uses the US dollar. Only a small number of the products were made within country; the products produced within New Caledonia were mainly bread, dairy and convenience meals, in Guam dairy and bread, whereas in Samoa they were mostly beverages, canned fish and chips/crisps made from local foods. Fiji had a diverse range of products made locally including sauces, dairy, drinks, snacks and biscuits. Overall foods were found from 54 countries, from as far as South and North America, Europe, Middle East and Asia (Figure 1).

The number of products with no nutritional information was highest in New Caledonia (14.4%), while all products in Nauru included a nutrient information panel of some type. The packaged foods with no nutrient information were from multiple origins, although in Guam many (n=28) were local (breads and cakes), and also in New Caledonia 95 were local (mostly breads, snacks, meats and beverages). The breadth of the nutrition information varied (Table 2). For example in New Caledonia, 27% of products (n=363) had no sodium information, whereas in Guam only 9 products had no sodium information (in addition to those with no nutrition information). Samoa, Nauru and Fiji were similar to Guam, with few products which had some nutrition information, but not sodium. Overall 51% (n=3387) of the products had data on trans fats regionally, with almost all products in Guam containing this information. By comparison, only 20% of products in Fiji contained trans fat information (n=288), and even less in New Caledonia, Nauru, and Samoa. Over 80% (n=4860) of the foods contained information on saturated fat content (n= 1181 without saturated fat data), while 826 products (14% of dataset) provided no saturated fat content but did provide other nutrition information such as energy and total fat. More than half of the foods (n=681) which contained no saturated fat content were from the New Caledonia data. Of these 48% (n=327) originated from France, and 83% were from other European countries (n=565).

**Table 2:** **Overview of the number of products with nutrient information panels which had sodium**, **trans fat or saturated fat data missing**

	**Fiji (n=1374)**	**Guam (n=2063)**	**Nauru (n=234)**	**New Caledonia (n=1140)**	**Samoa (n=875)**
Products with no sodium information on nutrient panel	106	9	23	363	57
Products with no trans fat information on nutrient panel	1086	22	208	1076	727
Products with no saturated fat information on nutrient information panel	246	19	73	681	262

All the data from products originating in the US had nutrient information given per serve only, as required by the food labelling legislation in the US. Nearly all other products provided data per 100 g (with some items providing per serve as well, but only the 100 g data was recorded), which is consistent with the labelling legislation in Australia and New Zealand. Conversion to per 100 g was problematic for products with small serve size declared. This included a spray cooking oil, which consisted of vegetable oil in a spray can. The serve size was only 0.5 ml, and was thus labelled as containing no fat or energy per serve. This could therefore not be converted to per 100 g. Checking of nutrient content identified 132 products with questionable values. These originated from twenty one countries, including the US (n=35) and Fiji (n=15). All product categories were included within this group. Examples include a sweet biscuit from China purporting to contain 92.5 g carbohydrate, 11.5 g protein and 17.4 g fat per 100 g. Two candy products from China were labelled as containing 130 g sugars per 100 g. Two vinegars from the US indicated they had zero energy content, but 6.3 to 12.2 g carbohydrate per 100 ml respectively.

**Figure 2:**
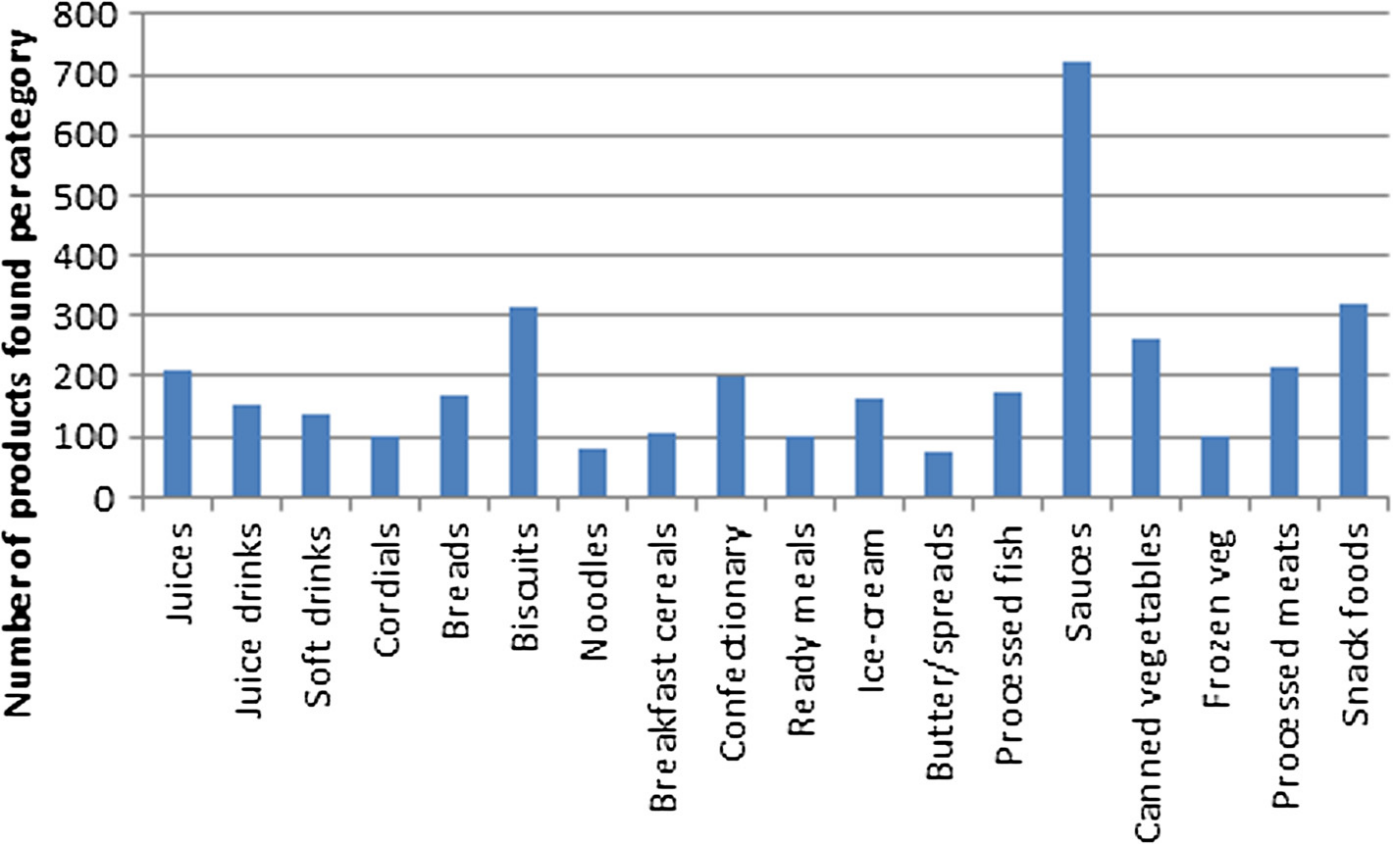
**Number of items from key food sub**-**categories in the region.**

In some product categories there were a considerable number of different products available. For example, with soft drinks (defined as sugar-sweetened or artificially sweetened non-alcoholic beverages), 83 varieties were available in New Caledonia, but only 27 in Samoa. In the snack food category which included crisps, extruded snacks and corn chips, Fiji had over 150 products, while Nauru had just 9 products. Figure 2 shows a selection of the food categories, and indicates that sauces, biscuits and snack foods had the largest variety at a regional level. Processed fish consisted mostly of canned fish, and it is a popular item in the region.

In many of the product categories there was a considerable range of nutrient levels reported, particularly for sodium, sugar and fat. For example in sausages there was more than a twofold difference in mean fat content of sausages between Nauru (10 g fat/100 g) and Guam (23 g fat/100 g) (Table 3). For tomato sauce, sugars contents varied almost tenfold (Table 4), with much smaller ranges available in Nauru and Samoa, and higher minimum values. Canned tuna, which is another popular item in the region, had fat contents ranging from <1 g to 20 g per 100 g, and sodium values ranging from 218 mg to 624 mg (13 products did not declare sodium content). The highest fat content was for a product described as canned in oil (from Spain), and the highest sodium item was a curried tuna product (from Thailand). The lowest sodium product was from Fiji, the lowest fat contents from Thailand and New Zealand.

**Table 3:** Fat content in sausages by country

	**Fiji**	**Guam**	**Nauru**	**New Caledonia**	**Samoa**
Number of sausage products recorded	24	24	3	0	8
Mean fat (g/100 g)	15	23	10		18
Lowest fat content (g/100 g)	7	7	5		11
Highest fat content (g/100 g)	25	45	14		26

**Table 4:** **Sugars content of tomato sauce**/**ketchup by country**

	**Fiji**	**Guam**	**Nauru**	**New Caledonia**	**Samoa**
Number of products recorded	11	15	4	8	3
Mean sugars content (g/100 g)	19	14	22	16	21
Lowest sugars content (g/100 g)	4	3.2	20	9	16
Highest sugars content (g/100 g)	30	24	23	23	25

Saturated fat content was high in products such as butter and coconut. A number of vegetable oils (labelled as such) containing 40-46 g saturated fat per 100 ml of product, originated from Malaysia and Indonesia, and were likely palm oil based. Some of the data for saturated fat content was extremely high, although potentially inaccurate, for example dried fried onions which contained 50 g/100 g (from Thailand), chocolate coated biscuits with 40 g/100 g (from Brazil) and an Indian-style spicy snack, chevda, with 33.9 g/100 g (from Fiji). In the biscuit category, ten products had saturated fat content declared as greater than 20 g/100 g. Of these two were from the United Arab Emirates, three from Dubai and three from New Zealand. In the extruded snack category, six of the seven highest for saturated content (16–19.8 g/100 g) were made in Fiji, whereas for potato crisps five of the six products (14.7-15.6 g/100 g) with the highest saturated fat content were from New Zealand, with the sixth from Fiji.

In the snack food category, nearly 10% of food products had labels with no nutrition information. Fat contents ranged from 0 g/100 g (rice crackers from India) to 52 g (Cheese balls from Malaysia). While 17 of the products declared 0 g sugars, the highest sugars content was 78 g/100 g for honey flavoured popcorn from Australia. The second highest were mini asparagus sticks from Japan, containing 60.7 g sugars/100 g.

Over one-quarter of the products contained some type of health-related claim or statement on the label, in addition to the nutrition panel (Table 5), with some including multiple claims. Statements claiming reduced fat or fat-free were found on 342 food items and claims that the food was a source of specific vitamins was found on 214 foods. Colour and flavouring-free claims were also popular, with 204 products specifying that they were free of artificial colours and flavours. Other claims included 'natural’, fortified/enriched, lactose-free, halal, caffeine-free, no high-fructose corn syrup, egg-free, milk-free, source of omega-3s, easy-to-digest, high-in-fibre, wholegrain and free of hydrogenated fats.

**Table 5:** **Health**-**related claims**

	**Number**
Number of products indicating some health-claim	1574
Claim related to fat content	342
Vitamin source	214
No artificial colours	208
No preservatives	194
Claim related to sugar content	167
Gluten-free	125
Natural	102
Cholesterol free	88
Source of iron	53
No monosodium glutamate	53

## Discussion

This survey illustrated the globalisation of processed food sources for the Pacific Islands, with foods available from every corner of the globe. There appears to have been a considerable increase in the number of countries of origin between 2008 and 2011. While the 2008 study design [8] was somewhat different from this study, it included data collection in 15 countries from the region. Also, this study found that relatively little of the packaged foods were made within the country or region, which can make it more difficult to control or influence the food supply. This reflects the low level of manufacturing in the region, with only a few countries having food processing plants, and Fiji having the largest number. The countries are heavily import dependant [1]. Available evidence suggests that the increasing use of imported and processed foods in the region has mirrored increases in non-communicable diseases [1,3,10].

There was a considerable range of processed foods sold within the countries, although far less than was found in similar surveys in the United Kingdom [11] and Australia [6]. The variety of foods sold did not mirror country size or extent of urbanisation [12], for example Fiji has the largest population out of the countries studied, and yet Guam had the largest product availability. Nauru has the highest levels of urbanisation, and yet the smallest number of processed foods.

Overall the extent of nutrition labelling was high, although the presence of perceived inaccurate labelling is of concern, as this can mislead consumers. The use of the per serve system of labelling nutrients, which is used principally in the US was problematic for this survey, as calculations were then needed to convert to per 100 g/100 ml to allow comparison with the products which were labelled per 100 g/100 ml. One significant issue with this were the errors introduced due to 'round-up’ and 'zero value’ definitions. This was particularly a problem with low declared serve sizes. In Fiji, legislation [13] allows for per serve or per 100 g nutrient information, this results in both styles of labelling be available within the country. This is not only a problem from a research perspective, but must also be problematic for consumers with limited understanding of interpreting processed food labels.

A related problem was found with products that require the addition of water before consumption, such as instant noodles, dried soups and other hot beverages. There was inconsistency in the nutrient panels, with some providing information only for diluted values, some only for the package as sold, and some providing both. For the purposes of this database, the requirement was for nutrient content in the product as sold. This necessitated calculating based on indicated volume of water to add, the nutrient content before dilution. In some cases information on the volume of water to be added was not provided, which raises the question of how the diluted nutrient values were calculated.

While the majority of products did have a nutrient information panel, these were often 'incomplete’ with key nutrients like sodium and trans fat missing. This presence/absence of certain nutrients was related to requirements of the legislation in the country of origin. For example Guam adheres to the food labelling standards of the US and therefore requires trans fat and sodium on the nutrient information panel. At the time of this study Fiji did not require these two nutrients to be on the nutrient information panel, although this has recently been added.

The range of salt, sugar and fat content in the foods and drinks found in the region is in line with similar studies elsewhere [6,11]. The informed consumer could reduce their intake of fats, sugars and sodium if they can interpret what is often incomplete labelling. There is clearly considerable scope for producers to reformulate products to improve their nutritional profile [14,15].

The use of health-related claims on the packaging indicates a willingness on the part of producers to promote their products from a nutrition or health perspective in their key markets. It is unknown if consumers in the region are influenced by these claims. The high use of claims that products are colouring or preservative-free is indicative of interest among consumers regarding the presence of these substances, along with monosodium glutamate and high-fructose corn syrup. These claims on labels can be of use to consumers [16] although use of words such as 'natural’ can be difficult to interpret meaningfully.

This study provides some very useful information. The database has been made available to individual participating countries, and a merged regional dataset has also been provided to nutritionists in other countries in the region. The datasets have also been provided to regional technical support organisations, allowing them to make use of these and assist in dissemination.

There were however some limitations to the study, including the selection of the countries from which to collect data and the reliance on food labels, rather than chemical analyses to provide nutrient levels. Further work is needed to compare this dataset with those of other countries; however this project has highlighted the need for global work with food producers to support reformulation, as it can be difficult otherwise for import-reliant countries, particularly, to change their food supply.

## Competing interests

The authors declare no competing interests.

## Authors’ contributions

WS and AR developed the proposal and data collection methodology based on international Collaborative group, and oversaw the data collection and analysis. ER, RLTG, KC, JF and CG undertook the sampling and oversaw data collection in each of their sites. WS led the project and drafted the manuscript. All authors read and approved the final manuscript.
